# Prevalence and laboratory analysis of malaria and dengue co-infection: a systematic review and meta-analysis

**DOI:** 10.1186/s12889-019-7488-4

**Published:** 2019-09-16

**Authors:** Manas Kotepui, Kwuntida Uthaisar Kotepui

**Affiliations:** Department of Medical Technology, School of Allied Health Sciences, Walailak University, Thasala, Nakhon Si Thammarat, Thailand

**Keywords:** Hematological parameters, Malaria prevalence, Dengue prevalence, Dual infection

## Abstract

**Background:**

A clear understanding of the epidemiology of malaria and dengue co-infection is essential for informed decisions on appropriate control strategies for dengue and malaria. This systematic review synthesized evidence on the relationship of malaria and dengue co-infection and related it to alterations in platelet, hemoglobin, hematocrit, aspartate aminotransferase (AST), and alanine aminotransferase (ALT) levels when compared to malaria mono-infection.

**Methods:**

A systematic review in accordance with PRISMA guidelines was conducted. All published articles available in PubMed and Web of Science (ISI) databases before October 21, 2017 were recruited. All epidemiological studies except case reports on the prevalence or incidence of malaria and dengue co-infection among patients visiting hospitals with febrile illness were included. Studies that involved conference abstracts, protocols, systematic reviews, only mono-dengue or mono-malaria infections, and only animal or in vitro studies were excluded after screening the titles, abstracts, and body texts. Studies were additionally excluded after full text review when they lacked epidemiologic data on malaria and dengue co-infection. Two reviewers independently screened, reviewed, and assessed all the studies. Cochrane Q (Chi-square) and Moran’s I^2^ were used to assess heterogeneity, and the funnel plot was used to examine publication bias. The summary odds ratio (OR) and 95% confidence intervals (CI) were estimated using a fixed-effects model. Thirteen cross-sectional and two retrospective studies were eligible to be included in the systematic review and meta-analysis.

**Results:**

Out of the 2269 citations screened, 15 articles were eligible to be included in the systematic review and meta-analysis. The 15 studies involved 13,798 (10,373 cases with malaria and 3425 with dengue) patients in 9 countries. Thirteen studies compared the incidence and odds of *Plasmodium* sp. infection, five studies compared the odds of mean platelet, three studies compared *Plasmodium* parasite density, and four studies compared the odds of hemoglobin, hematocrit, AST, and ALT levels among co-infected groups and single-malaria-infected groups.

**Conclusions:**

This study showed that dengue and malaria co-infection was associated with decreased odds of malaria infection, malaria parasitemia, AST, and ALT levels when compared to malaria mono-infection. However, malaria and dengue co-infection was associated with increased odds of platelet and hemoglobin levels when compared to malaria mono-infection.

**Electronic supplementary material:**

The online version of this article (10.1186/s12889-019-7488-4) contains supplementary material, which is available to authorized users.

## Author summary

A clear understanding of the epidemiology of malaria and dengue co-infection is essential for informed decisions on appropriate control strategies for both malaria and dengue. In this systematic review and meta-analysis, prevalence/incidence of malaria and dengue infection was related to differences in parasite density, hemoglobin, hematocrit, platelet count, and liver enzymes; AST and ALT among patients were synthesized. All published articles available in PubMed and Web of Sciences (ISI) before October 21, 2017 were searched. We found thirteen cross-sectional and two retrospective studies eligible to be included in the systematic review and meta-analysis. A summarized analysis of the study findings showed that dengue and malaria co-infection was associated with decreased odds of malaria infection, malaria parasitemia, AST, and ALT levels when compared to malaria mono-infection. However, malaria and dengue co-infection was associated with increased odds of platelet and hemoglobin levels when compared to malaria mono-infection.

## Background

Malaria and dengue are common in tropical and sub-tropical areas of the world, causing a high rate of morbidity and mortality especially among children [1, 2]. In 2015, about 212 million people were infected with malaria and 429,000 were estimated to have died globally due to malaria infection [1]. Additionally, more than 390 million people required preventive treatment for dengue and close to 96 million manifested clinical symptoms associated with severe dengue annually [2]. *Plasmodium* sp. can infect humans and manifest a wide range of signs and symptoms ranging from asymptomatic malaria to severe malaria [3]. Cerebral malaria, hypoglycemia, pulmonary edema, bleeding, acidosis, severe anemia, and acute renal failure were the major complications of severe malaria, which may result in death if no prompt or effective treatments are administered [3]. However, people living in endemic areas of malaria usually show asymptomatic or some non-specific symptoms such as fever, fatigue, chills, and malaise [4]. In the endemic areas of *P. falciparum* malaria, children up to 5 years of age had more common cases than older children and adults. This might be due to older children and adults receiving partial immunity from the infection [4, 5]. As mosquitoes are usually present in a tropical country, the co-infection of both malaria and dengue is evident and can cause acute febrile illness among patients. Atypical lymphocytosis, hemoconcentration, and thrombocytopenia are specific markers of dengue infection, which help differentiate the diagnosis of dengue infection from malaria infection [6–8].

A clear understanding of the epidemiology of malaria during dengue co-infection is essential for informed decisions on appropriate control strategies for dengue and malaria. In addition, we do not know the severity of co-infections when compared to single infections. The outcomes of co-infections are distinct among studies, especially in the selection criteria and diagnostic methods used in each study. Hence, the aim of this study was to perform a systematic review and meta-analysis to quantify the odds of *Plasmodium* infection, parasite density, and malaria-related alterations in hemoglobin, hematocrit, platelet, AST, and ALT levels among co-infected patients and mono-infected patients.

## Methods

### Search methods for identification of studies

The protocol for this systematic review and meta-analysis was conducted following the PRISMA guidelines (Checklist S1) [9], and the previous study reported the relationship of *S. haematobium* or *S. mansoni* and *P. falciparum* malaria infection [10]. Two authors (MK and KU) independently conducted a search in PubMed and Web of Science (ISI) databases using the keywords: “Plasmodium” OR “malaria” in combination with “dengue” (Additional file 1: Table S2) for articles published before October 21, 2017. The search was limited to human cases and to articles in the English language. Duplicates, abstracts, and titles were excluded from this study. A total of 2269 papers were screened for eligibility criteria, and 45 papers were chosen for full text evaluation. The discrepancies of choosing papers in this review were judged by a third reviewer. However, there was a very low degree of discrepancy between the two authors in this report.

### Eligibility criteria

All epidemiological studies which reported prevalence or incidence of *Plasmodium* sp. infection and dengue infection were included. Unpublished studies, case studies, conference abstracts, protocols, systematic reviews, and studies that involved only animal or in vitro studies were excluded after screening the titles and abstracts. Studies were additionally excluded following full text review if they lacked epidemiologic data on *Plasmodium* and dengue co-infection.

### Outcome measures

The primary outcome was prevalence/incidence of *Plasmodium* and dengue co-infection. Malaria was defined as microscopic confirmation of the *Plasmodium* parasite in blood without signs or symptoms of severe malaria. The secondary outcomes included parasite density, hemoglobin, hematocrit, platelet, AST, and ALT levels.

### Data extraction and management

Information about the authors, study area, study design, sample size enrolled, age range, prevalence of malaria and dengue co-infection, diagnosis techniques, and the main findings on prevalence/incidence of *Plasmodium* infection related to parasite density, hematocrit, platelet, AST, and ALT levels were abstracted and entered into an Excel sheet.

### Assessment of reporting biases

Quality and risk of bias of the studies was evaluated using the Effective Public Health Practice Project [11]. The quality of the studies was assessed on the basis of selection of the study participants, study design, confounder, blinding, data collection methods, withdrawals, and drop-outs comparability.

### Data synthesis

Heterogeneity was assessed using Cochrane Q (Chi-square) and Moran’s I^2^ (Inconsistency) using RevMan 5 software (Version 5, London, UK) [12]. Publication bias was evaluated using a funnel plot [13]. Odds ratio and mean differences along with the 95% confidence intervals were used as effect measures. The 95% CI for mean differences in parasite density, hemoglobin, hematocrit, platelet, AST, and ALT levels among those co-infected with dengue and those uninfected with dengue for the studies by Magalhaes et al. [14] and Mendonça et al. [15] were estimated using the mean and standard deviations values. The mean and standard deviations of hemoglobin, hematocrit, platelet, AST, and ALT levels for Mendonça et al. [15] and Assir et al. [16] were estimated from the median and interquartile range based on the formula suggested by Higgins et al. [17]. A fixed-effects model was used to estimate the summary Mantel-Haenszel odds ratio of malaria infection among patients infected with dengue and those uninfected with dengue.

## Results

### Search results and study characteristics

**Fig. 1:**
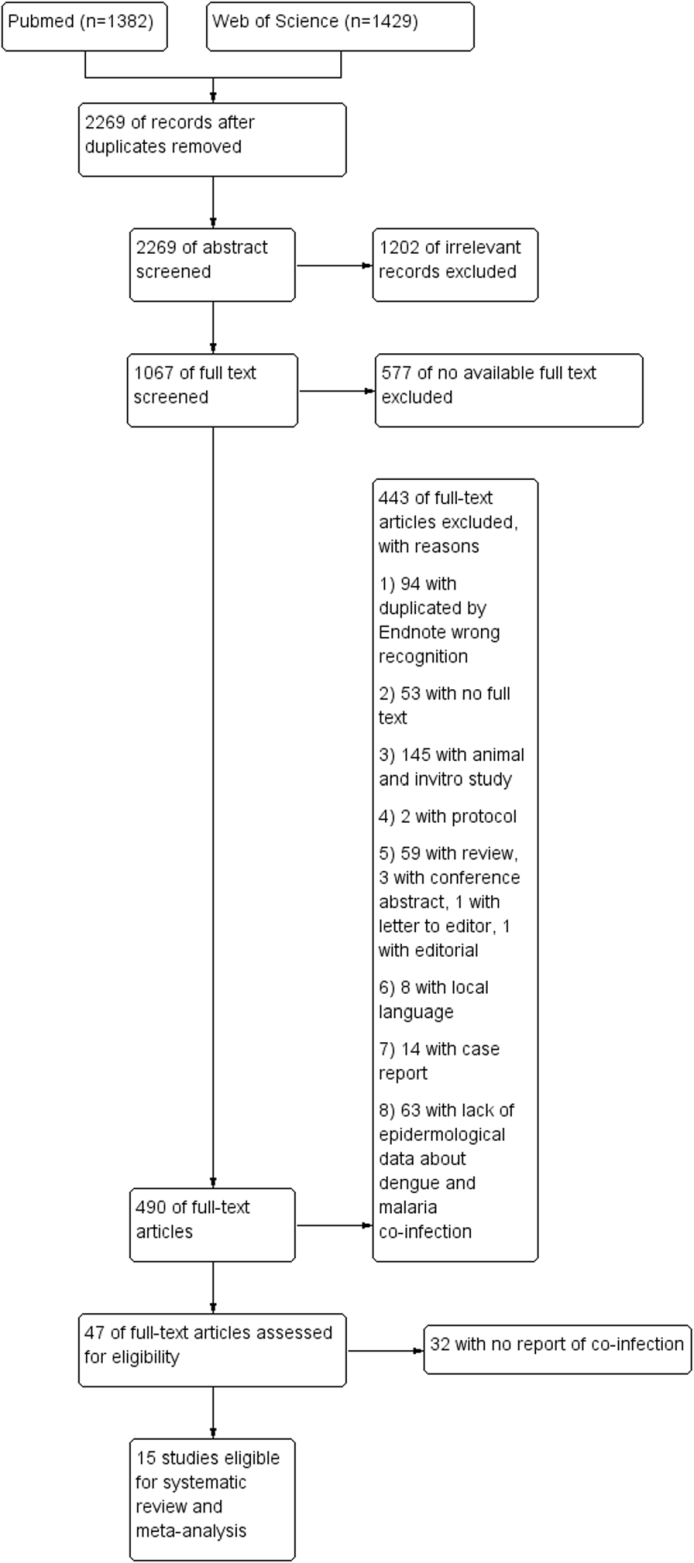
PRISMA diagram. Flow chart for study selection

A total of 2811 citations were identified from PubMed (*n* = 1382) and Web of Science (*n* = 1429) databases, of which 542 articles were found to be duplicates. Of the 2269 articles screened, there were 1202 articles excluded after reading the titles and abstracts due to irrelevant records. Of the 1067 articles screened thereafter, 577 articles were excluded due to a lack of full text. Of the 490 full text articles reviewed, 443 were excluded. Of the 47 full text articles reviewed, 32 articles were excluded due to a lack of information on co-infection. A total of 15 articles were considered for the systematic review and meta-analysis (Fig. 1). The characteristics of the 15 studies with 13,798 subjects (10,373 cases with malaria and 3425 with dengue) included in this review were summarized in Table 1. Thirteen studies were cross-sectional and two studies were retrospective.

**Table 1:** Characteristics of the included studies

Reference	Study area (years of the survey)	Age range	*Plasmodium* sp.	The magnitude of outcome in those co-infected compared to those with only malaria	Case enrolled	Case of co-infection	Case of malaria alone	Case of dengue alone	Diagnostic Techniques
Assir et al., 2014 [16]	Pakistan (2012)	12–90	*P.falciparum* and *P.vivax*	1. Co-infection rate = 1.99% 2. Hemoglobin = similar 3. Hematocrit = similar 4. Platelet count = similar	52	17	18	5	Malaria: Microscopy Dengue: ELISA NS1,IgM, RT-PCR
Baba et al., 2013 [18]	Nigeria (2008)	< 1- > 80 y	*P. falciparum*	1. Co-infection rate 7.17%	310	18	31	175	Malaria: Microscopy Dengue: Plaque reduction neutralization test (PRNT)
Barua and Gill, 2016 [19]	India (2014)	> 12 y	*P.falciparum* and *P.vivax*	1. Co-infection rate 10.25% 2. Hemoglobin = higher 3. Hematocrit = similar 4. Platelet count = lower 5. AST = higher 6. ALT = higher	156	16	55	85	Malaria: Microscopy Dengue: IgM, NS1
Carme et al., 2009 [20]	French Guiana (2004–2005)	NA	*P.falciparum* and *P.vivax*	1. Co-infection rate 1%	1723	17	376	221	Malaria: Microscopy Dengue: IgM serology, culture, RT-PCR
Chipwaza et al., 2014 [21]	Tanzania (2013)	2–13	NA	1. Co-infection rate 8.5%	364	31	52	45	Malaria: Microscopy Dengue: ELISA
Epelboin et al., 2012 [22]	French Guiana (2004–2010)	6 m-83 y	*P.falciparum* and *P.vivax*	1. Parasitemia = similar 2. Hemoglobin = similar 3. Hematocrit = lower 4. Platelet count = lower 5. AST = similar 6. ALT = similar	Not given	104	208	104	Malaria: Microscopy Dengue: Cell-culture virus isolation, RT-PCR, NS1, IgM, IgM, IgM + IgA
Hati et al., 2012 [23]	India (2005–2010)	NA	*P.falciparum* and *P.vivax*	1. Co-infection rate 1.5%	2971	46	194	559	Malaria: Microscopy Dengue: ELISA
Magalhaes et al., 2014 [14]	Brazil (2009–2011)	0- > 60 y	*P.falciparum* and *P.vivax*	1. Co-infection rate = 3.16% 2. Parasitemia = similar 3. Hematocrit = higher 4. Platelet count = lower 5. AST = higher 6. ALT = higher	1578	44	176	584	Malaria: PCR. Dengue: IgM, NS1, RT-PCR
Mendonça et al., 2015 [15]	Brazil (2009–2013)	IQR20.8–52.25 y	*P. vivax*	1. Parasitemia = similar 2. Hemoglobin = similar 3. Hematocrit = similar 4. Platelet count = similar 5. AST = lower 6. ALT = higher	Not given	30	52	30	Malaria: Microscopy, PCR Dengue: RT-PCR
Mohapatra et al., 2012 [24]	India (2011)	NA	*P.falciparum* and *P.vivax*	1. Co-infection rate = 6% 2. Parasitemia = lower 3. Hemoglobin = higher 4. Platelet count = lower 5. AST = lower 6. ALT = lower	469	27	102	340	Malaria: Microscopy Dengue: RDT (NS1, IgM)
Mueller et al., 2014 [25]	Cambodia (2008–2010)	7–49 y	*P.falciparum* and *P.vivax*	1. Co-infection rate = 3.24%	1475	27	727	53	Malaria: RDT, Nested-PCR Dengue: RT-PCR
Rao et al., 2016 [26]	India (2013)	< 1- > 15 y	*P.falciparum* and *P.vivax*	1. Co-infection rate = 1%	1980	22	229	723	Malaria: Microscopy, RDT. Dengue: Dengue NS1, ELISA, RT-PCR
Swoboda et al., 2014 [27]	Bangladesh (2007–2010)	≥8 y	*P.falciparum* and *P.vivax*	1. Co-infection rate = 0.76%	659	5	362	35	Malaria: Microscopy, RDT. Dengue: ELISA IgM
Sow et al., 2016 [28]	Senegal (2009–2013)	> 1 y	*P. falciparum*	1. Co-infection rate = 0.01%	13,845	1	7386	2	Malaria: Microscopy, RDT Dengue: RT-PCR
Zaki and Shanbag, 2010 [29]	India (2005)	1 m-12 y	*P.falciparum* and *P.vivax*	1. Co-infection rate = 0.33%	602	2	33	79	Malaria: Microscopy Dengue: ELISA

Thirteen studies can be used to compare the odds of *Plasmodium* sp. infection. These studies included: Assir et al. [16], Baba et al. [18], Barua and Gill. 2016 [19], Carme et al. [20], Chipwaza et al. [21], Hati et al. [23], Magalhaes et al. [14], Mohapatra et al. [24], Mueller et al. [25], Rao et al. [26], Swoboda et al. [27], Sow et al. [28], Zaki and Shanbag 2010. [29].

Five studies had data related to platelet count [14–16, 19, 24]. Three studies had data related to *Plasmodium* parasitemia [14, 15, 24]. Four studies had data related to hemoglobin, hematocrit, AST, and ALT levels [14, 15, 19, 24]. A study by Epelboin et al. [22] reported the incidence of those parameters in terms of percentages (without mean or median); therefore, this study was excluded from the meta-analysis.

### Prevalence of dengue and malaria infection

**Fig. 2:**
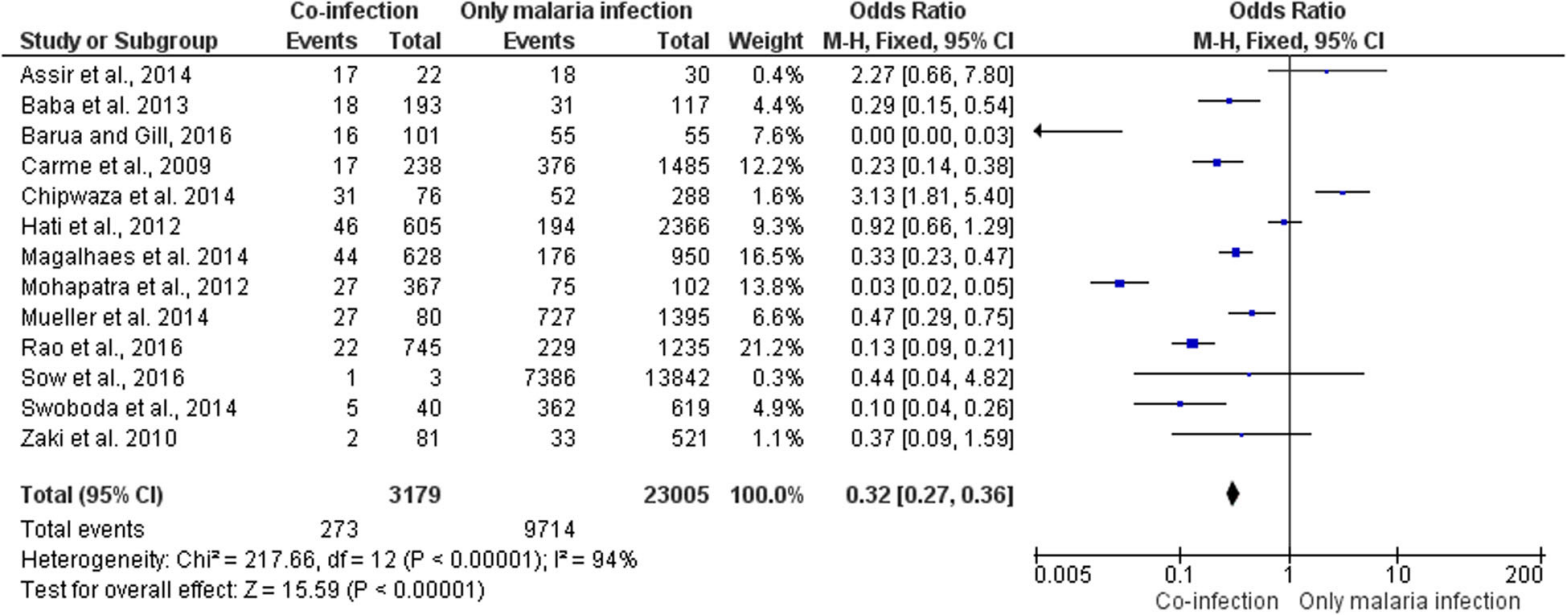
Forest plot showing the difference in the prevalence of malaria and dengue co-infection and those of malaria mono-infection

Thirteen studies examined the relationship of dengue infection with the odds of *Plasmodium* infection. Based on the meta-analysis shown in Fig. 2, cross-sectional studies in Nigeria [18], India [19, 24, 26], French Guiana [20], Brazil [14], Cambodia [25], and Bangladesh [27] showed significantly lower odds of co-infection when compared to those uninfected with dengue (OR: 0.29; 95% CI = 0.15, 0.54). A cross-sectional studies in Pakistan [16], Senegal [28], and India [23, 29] showed no significant odds of co-infection when compared to those uninfected with dengue (OR: 2.27; 95% CI = 0.66, 7.80). However, a study in Tanzania [21] showed significantly higher odds of co-infection when compared to those uninfected with dengue (OR: 3.13; 95% CI = 1.81, 5.40). The overall estimates based on thirteen studies showed significantly lower odds of *Plasmodium* infection among patients infected with dengue than those uninfected with dengue (summary OR: 0.32; 95% CI: 0.27, 0.36; I^2^: 94%) [14, 16, 18–21, 23, 25–29].

### Dengue infection and *Plasmodium* parasite density

**Fig. 3:**
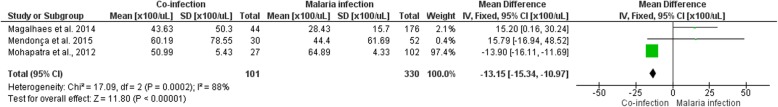
Forest plot showing the difference in parasitemia level of malaria and dengue co-infection and those of malaria mono-infection

Out of the fifteen studies included in this systematic review, three studies reported data in regard to the percent of the parasitemia of *Plasmodium* sp. Among these three studies, a study in the Brazilian Amazon reported co-infection with higher levels of parasitemia when compared to those uninfected with dengue (mean 4363 vs 2843 parasites/mm^3^) [14]. Another study in the Brazilian Amazon also showed a higher parasitemia level in patients with co-infection when compared to those uninfected with dengue [15]. However, a study in India [24] showed a lower parasitemia level in patients with co-infection when compared to those uninfected with dengue (mean 5098.8 vs 6489.4 parasites/mm^3^). A summary analysis based on these three studies showed significantly lower odds of *Plasmodium* parasitemia in patients co-infected with dengue as compared to those uninfected with dengue (summary mean difference = − 13.15; 95% CI = − 15.34, − 10.97; I^2^ = 88%) (Fig. 3) [14, 15, 24].

A study in French Guiana showed a lack of data in the mean or median *Plasmodium* parasitemia in infected patients; however, the proportion of patients with low parasitemia (proportion = 19.2%) was higher when compared to those uninfected with dengue (proportion = 11.5%), but the data was not statistically significant (*p* = 0.08) [22].

### Status of co-infection and hemoglobin level

Four studies reported data in regard to the hemoglobin level of co-infection with dengue and *Plasmodium*. A study by Assir et al. reported no difference in the hemoglobin level between patients with co-infection and those uninfected with dengue (median 13.0 vs 12.5 g/dL, *P* value = 0.09) [16]. A study by Barua and Gill [19] reported a significantly higher hemoglobin level in patients with co-infection than those uninfected with dengue (mean 8.5 vs 7.4 g/dL, *P* value < 0.001). A study by Mendonça et al. reported no significant difference in the hemoglobin level between patients with co-infection and those uninfected with dengue (median 13.0 vs 13.2 g/dL, *P* value = 0.48) [15]. Another study by Mohapatra et al. reported a significantly higher hemoglobin level in patients with co-infection than those uninfected with dengue (mean 10.7 vs 6.8 IU/L, *P* value = 0.001) [24].

**Fig. 4:**
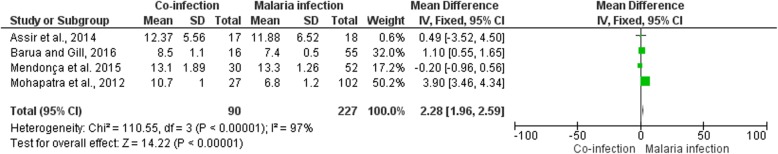
Forest plot showing the difference in hemoglobin level of malaria and dengue co-infection and those of malaria mono-infection

A summary analysis based on these four studies showed no significant differences in the odds of hemoglobin level between patients co-infected with dengue and those uninfected with dengue (summary mean difference = − 0.43; 95% CI = − 1.39, 0.53; I^2^ = 22%) (Fig. 4) [15, 16, 19, 24]. A study in French Guiana showed that the proportion of co-infected patients with low hemoglobin (< 12 g/dl) (proportion = 35.6%) was not significantly different when compared to those uninfected with dengue (proportion = 20.7%) (*p* = 0.05) [22].

### Status of co-infection and hematocrit level

**Fig. 5:**
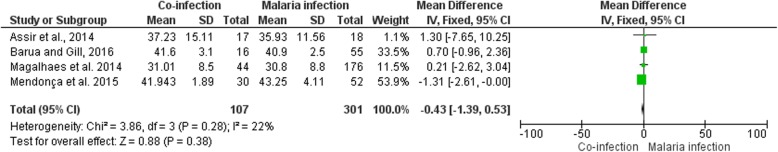
Forest plot showing the difference in hematocrit level of malaria and dengue co-infection and those of malaria mono-infection

Out of the fifteen studies included in this review, four studies reported data in regard to the hematocrit level of co-infection and that of *Plasmodium* sp. infection only. However, there was no difference found in the hematocrit level of patients with co-infection when compared to those uninfected with dengue (Magalhaes et al. mean 31.01 vs 30.8%, *P* value = 0.473; Mendonça et al. median 42.05 vs 43.35%, *P* value = 0.373; Assir et al. median 39.3 vs 36.0%, P value = 0.69; Barua and Gill mean 41.6% vs 40.9%) [14, 15]. A summary analysis based on these four studies also showed no significant difference in the odds of hematocrit level between patients co-infected and those uninfected with dengue (summary mean difference = − 0.43; 95% CI = − 1.39, 0.53; I^2^ = 22%) (Fig. 5). A study in French Guiana showed a lack of data in the mean or median hematocrit level in infected patients; however, the proportion of co-infected patients with low hematocrit (< 36%) (proportion = 54.3%) was significantly higher when compared to those uninfected (proportion = 23.6%) with dengue (*p* = 0.002) [22].

### Status of co-infection and platelet level

Out of the fifteen studies included in this review, five studies reported data in regard to the platelet level of co-infection and that of *Plasmodium* sp. infection only. A study by Assir et al. [16] in Pakistan reported no difference in platelets between patients with co-infection and those uninfected with dengue (median 54,000 vs 46,000/mm^3^, *P* value = 0.35). A study by Barua and Gill reported a significantly lower platelet level in those with co-infection when compared to those uninfected with dengue (mean 47,587 vs 76,422/mm^3^, *P* value < 0.001) [19].

A study by Magalhaes et al. in the Brazilian Amazon reported no difference in platelets between patients with co-infection and those uninfected with dengue (mean 69,772 vs 115,114/mm^3^, *P* value = 0.055) [14]. A study by Mendonça et al. in the Brazilian Amazon also reported no significant difference between patients with co-infection and those uninfected with dengue (median 87,500 vs 102,000/mm^3^, P value = 0.108) [15]. Another study by Mohapatra et al. reported no significant difference between patients with co-infection and those uninfected with dengue (median 58,230 vs 145,000/mm^3^, P value = 0.001) [24].

**Fig. 6:**
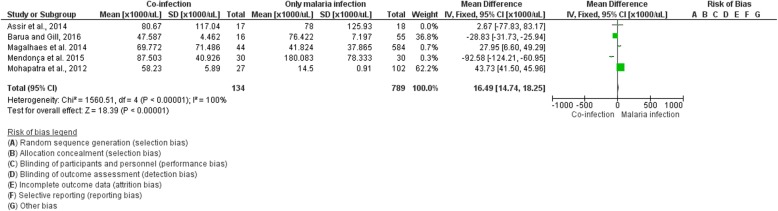
Forest plot showing the difference in platelet level of malaria and dengue co-infection and those of malaria mono-infection

A summary analysis based on these five studies showed significantly higher odds of platelets among patients co-infected with dengue as compared to those uninfected with dengue (summary mean difference = 16.49; 95% CI = 14.74, 18.25; I^2^ = 100%) (Fig. 6). A study in French Guiana showed a lack of data in the mean or median platelet level in infected patients; however, the proportion of co-infected patients with deep thrombocytopenia (< 50 g/L) (proportion = 23%) was significantly higher when compared to those uninfected (proportion = 6%) with dengue (*p* < 0.001) [22].

### Status of co-infection and AST level

Four studies reported data in regard to the AST level of co-infection with dengue and *Plasmodium*. A study by Barua and Gill in India reported a significantly higher AST level in patients with co-infection when compared to those uninfected with dengue (mean 116.3 vs 96.6 IU/L, *P* value < 0.001) [19].

**Fig. 7:**
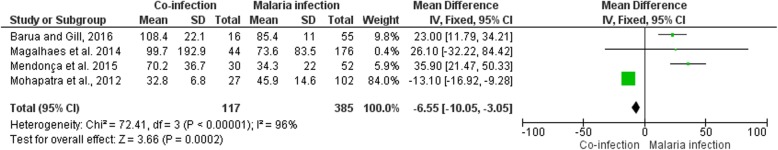
Forest plot showing the difference in AST level of malaria and dengue co-infection and those of malaria mono-infection

A study by Magalhaes et al. in the Brazilian Amazon reported no difference in the AST level between patients with co-infection and those uninfected with dengue (mean 90.9 vs 73.1 IU/L, P value = 0.263) (Fig. 7) [14]. However, another study by Mendonça et al. in the Brazilian Amazon reported a significantly higher AST level in patients with co-infection than those uninfected with dengue (median 47 vs 67.5 IU/L, P value = 0.005) [15]. A study by Mohapatra et al. reported a significantly lower AST level in patients with co-infection than those uninfected with dengue (mean 34 vs 51.7 IU/L, P value = 0.001) [24].

A summary analysis based on these four studies showed significantly lower odds of AST level in patients co-infected with dengue when compared to those uninfected with dengue (summary mean difference = − 1.6; 95% CI = − 14.24, − 8.96; I^2^ = 97%) (Fig. 6) [14, 15, 19, 24]. A study in French Guiana showed a lack of data in the mean or median AST level in infected patients; however, the proportion of co-infected patients with high AST (> 2 folds) (proportion = 10.2%) was not significantly different when compared to those uninfected with dengue (proportion = 16%) (*p* = 0.2) [22].

### Status of co-infection and ALT level

Four studies reported data in regard to the ALT level of co-infection with dengue and *Plasmodium*. A study by Barua and Gill in India reported a significantly higher ALT level in patients with co-infection when compared to those uninfected with dengue (mean 108.4 vs 85.4 IU/L, *P* value = 0.328) [19]. A study by Magalhaes et al. in the Brazilian Amazon reported no difference in the ALT level between patients with co-infection and those uninfected with dengue (mean 90.7 vs 73.6 IU/L, P value < 0.001) [14]. However, another study by Mendonça et al. in the Brazilian Amazon reported a significantly higher ALT level in patients with co-infection than those uninfected with dengue (median 69 vs 33 IU/L, P value < 0.0001) [15]. A study by Mohapatra et al. reported a significantly lower ALT level in patients with co-infection than those uninfected with dengue (mean 32.8 vs 45.9 IU/L, P value = 0.001) [24].

**Fig. 8:**
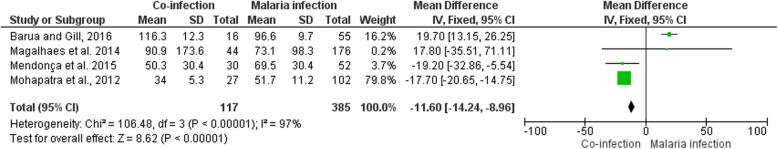
Forest plot showing the difference in ALT level of malaria and dengue co-infection and those of malaria mono-infection

A summary analysis based on these four studies showed significantly lower odds of ALT level in patients co-infected with dengue than those uninfected with dengue (summary mean difference = − 6.55; 95% CI = − 10.05, − 3.05; I^2^ = 96%) (Fig. 8) [14, 15, 19, 24]. A study in French Guiana showed that the proportion of co-infected patients with high ALT (> 2 folds) (proportion = 13%) was not significantly different when compared to those uninfected with dengue (proportion = 41%) (*p* = 0.16) [22].

### Quality of the studies

**Fig. 9:**
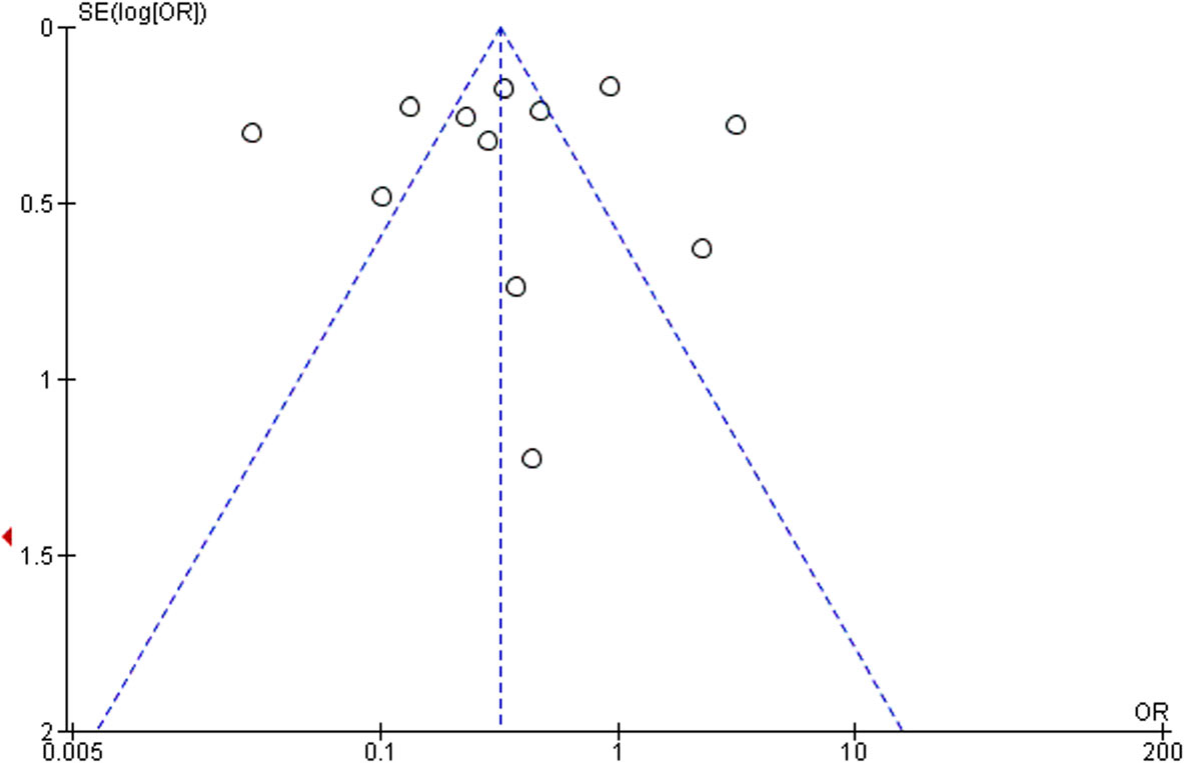
The funnel plot showed that there was no publication bias detected in the meta-analysis

Selection bias, study design, confounders, blinding, data collection methods, withdrawals, and dropouts according to the Effective Public Health Practice Project were summarized in Table 2 [11]. The majority of the studies showed strong quality in confounder and data collection methods. Most studies in this review were moderate in terms of selection bias. However, low quality in study design (cross-sectional study) and blinding were found in most of the studies. The overall rating based on the six criteria showed that none of the studies were of strong quality. Ten studies were of moderate quality and five studies were of weak quality. However, none of these studies were excluded from this review. The funnel plot showed that there was no publication bias detected in the meta-analysis (Fig. 9).

**Table 2:** Assessment of the quality of the studies included in the review based on Effective Public Health Practice Project: Quality assessment tool for quantitative studies

No.	Author, Year	Selection Bias	Study Design	Confounders	Blinding	Data collection method	Withdrawals and Drop-Out	Final Rating
1	Assir et al., 2014 [16]	3	3	1	3	1	NA	3
2	Baba et al., 2013 [18]	2	3	3	3	2	NA	3
3	Barua and Gill, 2016 [19]	2	3	1	3	1	NA	2
4	Carme et al., 2009 [20]	2	3	1	3	2	NA	2
5	Chipwaza et al., 2014 [21]	2	3	3	3	2	NA	3
6	Epelboin et al., 2012 [22]	2	3	1	2	1	NA	2
7	Hati et al., 2012 [23]	3	3	2	3	2	NA	3
8	Magalhaes et al., 2014 [14]	2	3	1	2	1	NA	2
9	Mendonça et al., 2015 [15]	2	3	1	2	1	NA	2
10	Mohapatra et al., 2012 [24]	2	3	2	3	1	NA	2
11	Mueller et al., 2014 [25]	2	3	3	2	1	NA	2
12	Rao et a., 2016 [26]	2	3	3	3	1	NA	2
13	Sow et al., 2016 [28]	2	3	2	3	1	NA	2
14	Swoboda et al., 2014 [27]	3	3	3	3	2	NA	3
15	Zaki SA and Shanbag P, 2010 [29]	2	3	3	3	3	NA	3

## Discussion

In the present systematic review of 13 studies based on 12,546 patients infected with malaria and/or dengue, a summary meta-analysis of these 13 studies confirmed decreased odds of co-infected patients as compared to those uninfected with dengue. The finding of a higher prevalence of *Plasmodium* co-infection with dengue could be due to both diseases sharing the same endemic regions [14]. In those areas (especially in rural, semi-urban, and urban areas), the vector *Anopheles* and *Aedes* are present throughout the year. Geographical overlap of both diseases exists for the 3.2 and 3.9 billion people who live in an endemic area for malaria and dengue, respectively [30, 31]. Therefore, the co-infection of both agents in a patient could not be ignored by physicians [26]. The co-infection of malaria and dengue had been reported in the Brazilian Amazon, Nigeria, India, French Guiana, and Tanzania [14, 18, 20, 21, 26, 32]. Infection by these two pathogens may share similar and non-specific clinical signs and symptoms – such as fever, headache, body ache, and fatigue – which may result in the difficulty of identifying one pathogen from the other [33]. A study indicated that the prevalence of co-infection was estimated among hospitalized patients but not in the community [14]. Another study in French Guiana reported that co-infection was due to the high rate of the population’s mobility to malaria endemic areas [20]. One study reported that co-infection frequency was higher especially during September to November [23]. Moreover, another study reported on an asymptomatic malaria infection with a low parasitemia course co-infection in an individual patient [34].

In regard to the immunity of individual patients, a previous study showed that co-infection has been associated with a strong activation of acute phase response, such as Interleukin 6 (IL-6), Tumor necrosis factor-α (TNF-α), and IL1-β and also Th1 cytokines (IFN-γ and IL-12). However, the lower levels of inflammation in the co-infected group were similar to DENV mono-infected subjects [33]*.* A co-infection exhibited positive TNF, IL-6, Interferon gamma (IFN-γ), IL-7, C-C Motif Chemokine Ligand 4 (CCL4), and IL-10 which was not observed in malaria mono-infection [15]. The co-infection may be caused by the DENV infection reactivating the hypnozoites of *P. vivax* in the liver, which were asymptomatic for months or years [33]. Previous studies that involved *P. falciparum* co-infection have reported fatalities [8, 35–38]. However, the frequency of severe clinical symptoms occurs in *P. vivax* co-infection [14, 22]. Those severe clinical symptoms may be caused by the activation of acute phase response including IL-6, TNF-α, IL1-β, IFN-γ, and IL-12 [33]. A previous study also showed that co-infection resulted in similar days of fever as compared to single malaria infection, which should therefore raise the suspicion of malaria co-infection [14].

The results from our meta-analysis found that co-infected patients exhibited lower malaria parasitemia than those with malaria single infection. This was in accordance with a previous study in French Guiana [22]. A previous study indicated that low parasitemia is a good predictive marker for less severe symptoms in co-infected patients [24]. The good outcome was because the concurrence of dengue and malaria led to patients seeking out medical treatment earlier (2.2 ± 0.4 days) than those with single malaria infection (5.5 ± 0.9 days), resulting in early diagnosis and treatment with antimalarial drugs [7, 36]. This was the frequency found in *P. vivax* co-infection [39–41].

Several studies reported on co-infection cases with severe anemia single malaria infection [14, 16, 22, 42]. Based on this meta-analysis, the hemoglobin level of co-infected patients were significantly higher than those with malaria single infection. This was in contrast to previous studies that indicated low hemoglobin in patients with co-infection [16, 42]. Malaria infection causes destruction of red blood cells followed by hemolysis and anemia [43]. Considering the clinical outcome, co-infection resulted in a lower rate of jaundice than those with dengue single infection [16].

Hematocrit is a marker used to diagnose dengue infection. Severe dengue results in hemoconcentration (the basal hematocrit > 20%), which is the result of increased vascular permeability and plasma leakage in endothelial [44]. Several studies reported no significant change in hematocrit among patients with co-infection when compared to those with malaria single infection [14–16, 19]. For malaria infection cases, low hematocrit was due to anemia, a common complication in both *P. falciparum* and *P. vivax* malaria [45]. However, this meta-analysis showed no significant odds in hematocrit level among those two groups of patients.

This meta-analysis found that co-infected patients had higher odds of platelet count as compared to malaria infected patients. This indicated that co-infected patients had a higher platelet level than malaria infected patients. Some studies indicated that co-infected patients also exhibited lower platelet count or thrombocytopenia than those with malaria single infection [15, 19, 22]. In regard to this meta-analysis, a significantly lower platelet level among co-infected patients was found in two studies [15, 19]. However, two other studies reported that malaria single infection exhibited thrombocytopenia more frequently than co-infection [14, 24]. For the clinical outcome of thrombocytopenia, co-infection had a higher chance of bleeding when compared to malaria single infection (OR 12.5, 95% CI: 4.7–33.3, *P* value = 0.001) [14].

AST is found in highest concentrations in the heart and also found in the liver, whereas ALT is found mainly in the liver [46]. Elevated AST and ALT can be seen in any type of liver cell injury [47]. Currently, the difference between liver enzymes (AST and ALT) and the clinical outcome of co-infection and malaria single infection is not well established. This meta-analysis found significantly lower levels of AST and ALT in co-infection when compared to malaria single infection. This indicated less liver injuries in the co-infected group. Liver injury was prominent in dengue single infection but not in malaria infection. A previous study showed that liver injuries and bleeding can lead to fulminant liver failure in dengue infection [48]. However, previous studies reported that hepatomegaly was very frequent in the co-infected group [14, 19]. Moreover, higher AST and ALT levels were associated with jaundice and hepatomegaly [14, 19]. Nevertheless, a report in French Guiana showed no differences in AST/ALT and parasitemia levels between co-infection and malaria single infection [22].

In terms of mortality, co-infection can lead to an increased mortality rate when compared to malaria single infection (6.3% compared to 5.5%) [19]. However, a study by Mohapatra et al. found a lower rate of mortality in co-infection as compared to malaria single infection [24]. The clinical outcome of co-infection was more similar to dengue single infection than malaria single infection. Therefore, the physician must be aware of co-infections in malaria cases with inadequate treatment response as well as screening for malaria parasite in patients with dengue [24].

The interaction of dengue and malaria in co-infections is unknown but the multiple infections may lead to a failure in treatment [49]. The underlying conditions in co-infections are rhabdomyolysis and sickle cell disease, which result from TNF-α and RBC sequestration in skeletal muscle, increased blood viscosity, and toxins from the parasite together with lactic acidosis [35]. A study found that co-infection had a predominance of Immunoglobulin M (IgM) antibody [22]. Another study indicated that malaria infection might be triggered by dengue infection, especially in the *P. vivax* infection [50].

This study had limitations. First, there was a high level of bias in the study design of the enrolled studies that were reviewed. Second, there was a high level of heterogeneity among the studies examining co-infection as compared to malaria single infection (Moran’s I^2^: 94%). Third, we lacked the access to some full text papers because our university does not subscribe to some publishers which reported on the co-infection of both agents.

Since malaria and dengue frequently co-exist in the same geographical areas, there are some public health implications. In addition, the clinical outcomes of co-infection were more like dengue mono-infection than malaria mono-infection. Therefore, healthcare workers including physicians, medical technicians, and nurses need to collaborate with each other in order to solve the difficulty of differentiating between both diseases in similar areas. Using clinical outcomes such as fever with typical paroxysm, cerebral malaria, renal failure, and multi-organ failure might rule out patients with co-infection. On the other hand, using bleeding signs might indicate patients with co-infection. Moreover, screening for malaria parasite in patients with dengue infection might help to diagnose patients suspected with co-infection [24].

## Conclusion

In conclusion, the study findings showed that dengue and malaria co-infection was associated with decreased odds of malaria infection, malaria parasitemia, AST, and ALT levels when compared to malaria mono-infection. However, malaria and dengue co-infection was associated with increased odds of platelet and hemoglobin levels when compared to malaria mono-infection.

## Additional file

Additional file 1: **Table S2.** Search details for the PubMed. (DOCX 13 kb)

## Data Availability

The datasets used during the current study are available from the corresponding author based on reasonable request.

## Abbreviations

ALT: Alanine aminotransferase
AST: Aspartate aminotransferase
CCL4: C-C Motif Chemokine Ligand 4
CI: Confidence intervals
DENV: Dengue virus
IFN-γ: Interferon gamma
IgM: Immunoglobulin M
IL-6: Interleukin 6
OR: Odds ratio
TNF-α: Tumor necrosis factor-α
