# Supplementary material for: Prevalence and laboratory analysis of malaria and dengue co-infection: a systematic review and meta-analysis
Source: BMC Public Health. 2019 Sep 16;19:1148. doi: 10.1186/s12889-019-7488-4 (PMC6745805; doi:10.1186/s12889-019-7488-4)
Supplement: Supplementary file 1 — Table S2. Search details for the PubMed. (DOCX 13 kb) [file 12889_2019_7488_MOESM1_ESM.docx]

Additional file 1: Table S2. Search details for the PubMed

| (("Plasmodium"[All Fields] OR "malaria"[All Fields]) AND ("dengue"[ All Fields]) |
| --- |
